# MAGNETIC SPHINCTER AUGMENTATION DEVICE FOR GASTROESOPHAGEAL REFLUX DISEASE: EFFECTIVE, BUT POSTOPERATIVE DYSPHAGIA AND RISK OF EROSION SHOULD NOT BE UNDERESTIMATED. A SYSTEMATIC REVIEW AND META-ANALYSIS

**DOI:** 10.1590/0102-672020230063e1781

**Published:** 2024-03-04

**Authors:** Agustin Cesar Valinoti, Cristian Agustin Angeramo, Nicolas Dreifuss, Fernando Augusto Mardiros Herbella, Francisco Schlottmann

**Affiliations:** 1 Hospital Aleman de Buenos Aires, Esophagus and Stomach Surgical Unit – Buenos Aires, Argentina; 2 Universidade Federal de São Paulo, Escola Paulista de Medicina, Surgery – São Paulo (SP), Brazil

**Keywords:** Gastroesophageal Reflux, Esophagus, Laparoscopy, Refluxo Gastroesofágico, Doenças do Esôfago, Esôfago

## Abstract

**BACKGROUND:**

Magnetic ring (MSA) implantation in the esophagus is an alternative surgical procedure to fundoplication for the treatment of gastroesophageal reflux disease.

**AIMS:**

The aim of this study was to analyse the effectiveness and safety of magnetic sphincter augmentation (MSA) in patients with gastroesophageal reflux disease (GERD).

**METHODS:**

A systematic literature review of articles on MSA was performed using the Medical Literature Analysis and Retrieval System Online (Medline) database between 2008 and 2021, following the Preferred Reporting Items for Systematic Reviews and Meta-Analyses (PRISMA) guidelines. A random-effect model was used to generate a pooled proportion with 95% confidence interval (CI) across all studies.

**RESULTS:**

A total of 22 studies comprising 4,663 patients with MSA were analysed. Mean follow-up was 27.3 (7–108) months. The weighted pooled proportion of symptom improvement and patient satisfaction were 93% (95%CI 83–98%) and 85% (95%CI 78–90%), respectively. The mean DeMeester score (pre-MSA: 34.6 vs. post-MSA: 8.9, p=0.03) and GERD-HRQL score (pre-MSA: 25.8 vs. post-MSA: 4.4, p<0.0001) improved significantly after MSA. The proportion of patients taking proton pump inhibitor (PPIs) decreased from 92.8 to 12.4% (p<0.0001). The weighted pooled proportions of dysphagia, endoscopic dilatation and gas-related symptoms were 18, 13, and 3%, respectively. Esophageal erosion occurred in 1% of patients, but its risk significantly increased for every year of MSA use (odds ratio — OR 1.40, 95%CI 1.11–1.77, p=0.004). Device removal was needed in 4% of patients.

**CONCLUSIONS:**

Although MSA is a very effective treatment modality for GERD, postoperative dysphagia is common and the risk of esophageal erosion increases over time. Further studies are needed to determine the long-term safety of MSA placement in patients with GERD.

## RESUMO

**RACIONAL:**

A implantação de anel magnético (AM) no esôfago é um procedimento cirúrgico alternativo à fundoplicatulra, para o tratamento da doença do refluxo gastroesofágico.

**OBJETIVOS:**

O objetivo deste estudo foi analisar a eficácia e segurança do anel magnético em pacientes com doença do refluxo gastroesofágico (DRGE).

**MÉTODOS:**

Uma revisão sistemática da literatura de artigos sobre AM foi realizada usando o banco de dados Medline entre 2008 e 2021, seguindo as diretrizes PRISMA. Um modelo de efeito aleatório foi usado para gerar uma proporção agrupada com intervalo de confiança (IC) de 95% em todos os estudos.

**RESULTADOS:**

Um total de 22 estudos compreendendo 4.663 pacientes submetidos à colocação do AM foram analisados. O seguimento médio foi de 27,3 (7–108) meses. A proporção ponderada de melhora dos sintomas e satisfação do paciente foi de 93% (IC95% 83–98%) e 85% (IC95% 78–90%), respectivamente. A pontuação média de DeMeester (pré-AM: 34,6 *versus* pós-AM: 8,9, p=0,03) e pontuação GERD-HRQL (pré-AM: 25,8 *versus* pós-AM: 4,4, p<0,0001) melhoraram significativamente após a colocação do anel. A proporção de pacientes em uso de inbidor de bomba de prótons (IBP) diminuiu de 92,8% para 12,4% (p<0,0001). A erosão esofágica ocorreu em 1% dos pacientes, o risco aumentou significativamente para cada ano de uso do AM (OR 1,40; IC95% 1,11–1,77, p=0,004). A remoção do dispositivo foi necessária em 4% dos pacientes.

**CONCLUSÕES:**

O AM é uma modalidade de tratamento eficaz para a DRGE. A disfagia pós-operatória é comum, e o risco de erosão esofágica aumenta com o tempo.

**Figure 1:**
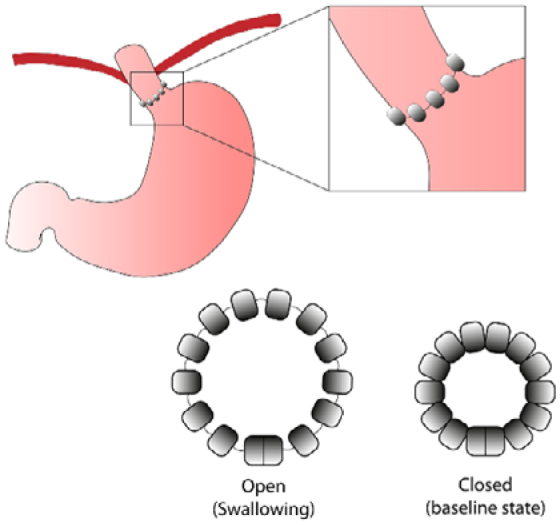
Location of the magnetic sphincter augmentation device.

## INTRODUCTION

Gastroesophageal reflux disease (GERD) is a condition which develops when the reflux of stomach contents into the esophagus causes troublesome symptoms or complications^53^. GERD is the most prevalent gastrointestinal disorder across the United States, ranging from 6 to 30%, with approximately 110 thousand hospital admissions annually^2,21,51^. Current evidence suggests that its incidence is increasing, mainly due to the rising prevalence of obesity worldwide^35^. As GERD significantly impairs quality of life and work productivity, it also represents a substantial financial burden to the health-care system^27^.

Dietary and lifestyle modifications along with antireflux medication (i.e. proton pump inhibitors [PPI]) are the mainstay of treatment for GERD. However, it is estimated that up to 40% of patients fail to respond to medical therapy^15,22,30,32,36^. The Society of American Gastrointestinal and Endoscopic Surgeons (SAGES) guidelines on the management of GERD recommend that surgical treatment be considered in individuals who have failed medical therapy, have GERD complications, and/or present extra-esophageal manifestations^39^.

The laparoscopic fundoplication has been the most common surgical procedure to treat GERD in the last decades^2,24,37^. In 2008, a multicenter study described a novel laparoscopically implantable magnetic sphincter augmentation (MSA) device designed to restore the lower esophageal sphincter barrier function^13^. It consists in multiple adjustable beads that are placed around the gastroesophageal junction. The magnetic union between each of the beads allows the passage of the swallowed bolus but inhibits the reflux of stomach contents into the esophagus^5^ (Figure 1). Although several studies have demonstrated the safety and efficacy of the device^3-5,7,10-14,16,19,20,23,25,26,29,32-34,40,41,43,45,46,48-50,52,55^, large series and randomized trials supporting its use are still lacking.

**Figure 1:**
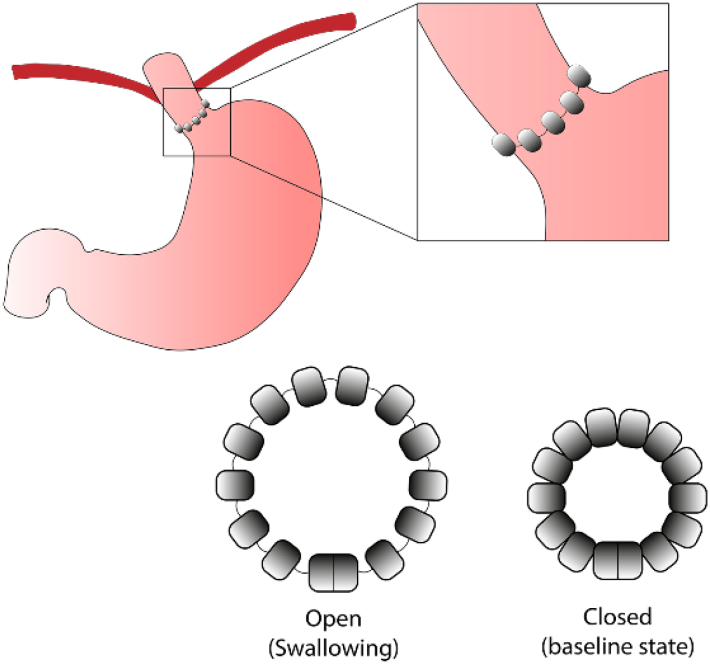
Schematic representation of the magnetic sphincter augmentation device placed around the gastroesophageal junction.

The aim of this systematic review and meta-analysis was to summarize all the currently available evidence on MSA to determine its safety and effectiveness for GERD treatment.

## METHODS

### Search strategy

A systematic literature review of articles on laparoscopic MSA device placement was performed according to the *Preferred Reporting Items for Systematic Reviews and Meta*-*Analyses* (PRISMA) guidelines. Medical Literature Analysis and Retrieval System Online (Medline) and Cochrane Central Register of Controlled Trials databases were systematically searched for all articles published from January 2008 to February 2021. The following medical subject headings were used to identify relevant studies: "magnetic sphincter augmentation", "LINX", "Magnetic sphincter augmentation and gastroesophageal reflux disease", "Magnetic sphincter augmentation for GERD". The keywords were used in all possible combinations to obtain the maximal number of articles. The reference list of the retrieved articles was also screened to find articles that were missed during the primary search.

### Selection criteria and data extraction

All studies reporting outcomes of patients who underwent MSA device implantation were included in the analysis. The search was limited to articles in English. Experimental studies in animal models, abstracts, case reports, reviews, editorials, and comments were excluded.

A total of 617 articles were initially screened; after removing duplicates and excluding titles and abstracts that did not meet the inclusion criteria, 52 articles were revised by two independent authors (ACV and CAA) based on the methodological quality of the publications. Discrepancies between the two reviewers were resolved by discussion and consensus with the senior author (FS). Finally, 22 articles were included for the meta-analysis^15-36^ (Table 1 and 2). The investigators (ACV, CAA) independently evaluated and extracted the data from all the eligible publications. The following data were extracted from the articles: author, publication year, design, population size, gender, age, body mass index (BMI), follow-up, preoperative GERD Health-Related Quality of Life (GERD-HRQL) score, preoperative use of proton pump inhibitor (PPI), preoperative DeMeester score, operative time, 30-day overall morbidity, 30-day mortality, length of hospital stay (LOS), symptoms improvement, satisfaction rates, postoperative GERD-HRQL score, postoperative use of PPI, postoperative DeMeester score, dysphagia, endoscopic dilation, gas-related symptoms, erosion, and device removal rates.

**Table 1:** Characteristics of studies included in the meta-analysis pre-magnetic sphincter augmentation.

Author	Year	Type of study	Patients (n)	Pre-MSA				
				Median follow-up time (months)	GERD- HRQL score	Use of PPI	DeMeester score	Median operative time (min)
Lipham et al.^32^	2012	R	44	44	25.7	100	NR	40
Lipham et al.^33^	2015	R	1048	44	NR	NR	NR	NR
Sheu et al.^49^	2015	R	12	7	NR	NR	NR	NR
Ganz et al.^25^	2016	P	100	60	27	100	NR	NR
Reynolds et al.^41^	2016	R	52	12	17	NR	NR	66
Rona et al.^45^	2017	R	140	20	18.6	NR	39.3	NR
			52	20	20.5	NR	52.4	NR
Czosnyka et al.^16^	2017	R	102	7.6	NR	NR	NR	49
Buckley et al.^14^	2018	P	200	8.6	26	87	NR	81
Prakash et al.^40^	2018	R	47	36	25.8	100	34.1	74
Louie et al.^34^	2019	P	200	12	26	NR	33.4	NR
Antiporda et al.^4^	2019	R	98	46	25	NR	33.8	NR
Tatum et al.^50^	2019	R	96	18.5	NR	NR	31.2	56.4
			86	12.5	NR	NR	19.9	69.3
Ward et al.^55^	2020	R	86	12	38.79	100	NR	NR
Tsai et al.^52^	2020	R	118	7.8	42.3	91.6	NR	NR
Riva et al.^43^	2020	P	45	12	19	66.7	NR	NR
Dunn et al.^20^	2020	R	87	35	20.5	NR	34	60
Ferrari et al.^23^	2020	R	124	108	NR	NR	NR	NR
			211	108	NR	NR	NR	NR
Dominguez-Profeta et al.^19^	2020	R	68	NR	NR	97.1	52.9	NR
Allman et al.^3^	2021	R	86	NR	NR	89.5	40.9	NR
			51	NR	NR	96.1	40.3	NR
Ayazi et al.^7^ *	2020	R	553	10.3	33.8	NR	33.9	NR
Bonavina et al.^11^ *	2021	P	465	36	22	97.8	NR	43
Schwameis et al.^48^	2021	R	274	13.6	NR	88.1	22.8	NR
			60	13.6	NR	93	79.2	NR

Notes:

Abbreviations: MSA: Magnetic sphincter augmentation; GERD-HRQL: Gastroesophageal reflux disease health-related quality of life; PPI: Proton pump inhibitors; NR: not reported; R: retrospective; P: prospective.

* Studies with two groups of individuals undergoing MSA procedure.

**Table 2:** Characteristics of studies included in the meta-analysis post-magnetic sphincter augmentation.

Author	Year	Type of study	Patients (n)	Post-MSA								
				Symptom improvement (%)	Patient satisfaction (%)	GERD- HRQL score	Use of PPI	DeMeester score	Dysphagia (%)	Endoscopic dilation (%)	Erosion (%)	Removal (%)
Lipham et al.^32^	2012	R	44	NR	87.5	3.3	20	NR	43	NR	0	6.8
Lipham et al.^33^	2015	R	1048	NR	NR	NR	NR	NR	1.7	5.6	0.1	3.4
Sheu et al.^49^	2015	R	12	NR	NR	NR	NR	NR	83	50	0	NR
Ganz et al.^25^	2016	P	100	NR	92.9	4	15.3	NR	6	NR	0	7
Reynolds et al.^41^	2016	R	52	NR	90	4	15	NR	46	19	NR	0
Rona et al.^45^	2017	R	140	91.3	NR	5.6	26.6	NR	NR	17.9	NR	2.1
			52	98.1	NR	3.6	9.6	NR	NR	13.5	NR	0
Czosnyka et al.^16^	2017	R	102	NR	NR	NR	NR	NR	NR	9	NR	1
Buckley et al.^14^	2018	P	200	NR	NR	2	6	NR	6	10	0	1
Prakash et al.^40^	2018	R	47	NR	83.3	4.5	16.7	NR	NR	4.2	NR	0
Louie et al.^34^	2019	P	200	NR	80	4	12.6	12	36.6	7.1	0.5	2.5
Antiporda et al.^4^	2019	R	98	NR	77	5	28	NR	9.2	13.3	1.02	5.1
Tatum et al.^50^	2019	R	96	NR	NR	NR	23.4	NR	67	16.3	NR	8.3
			86	NR	NR	NR	19	NR	55.3	15.5	NR	3.7
Ward et al.^55^	2020	R	86	NR	87	6.53	9	NR	NR	3.5	NR	5.8
Tsai et al.^52^	2020	R	118	NR	NR	5.3	4.8	NR	67.8	16.9	NR	2.5
Riva et al.^43^	2020	P	45	NR	NR	3	22	NR	53.7	NR	NR	NR
Dunn et al.^20^	2020	R	87	NR	NR	4	29.3	13.7	1.08	NR	NR	NR
Ferrari et al.^23^	2020	R	124	NR	NR	21	NR	31.3	0.8	0	0	2.4
			211	NR	NR	19.5	NR	24.8	2.4	0	2.8	13.3
Dominguez-Profeta et al.^19^	2020	R	68	NR	NR	NR	7.1	NR	47.1	22.1	NR	4.4
Allman et al.^3^	2021	R	86	100	NR	NR	4.4	NR	7.5	2.9	NR	NR
			51	98	NR	NR	10.2	NR	26.5	18.4	NR	NR
Ayazi et al.^7^ *	2020	R	553	84	NR	7.2	6.3	NR	16.8	30.5	0	6.7
Bonavina et al.^11^ *	2021	P	465	93.1	NR	4.6	24.2	NR	3.8	NR	NR	2.4
Schwameis et al.^48^	2021	R	274	NR	NR	NR	6.9	NR	14.2	32.8	NR	5.1
			60	NR	NR	NR	15	NR	10	25	NR	3.3

Notes:

Abbreviations: MSA: Magnetic sphincter augmentation; GERD-HRQL: Gastroesophageal reflux disease health-related quality of life; PPI: Proton pump inhibitors; NR: not reported; R: retrospective; P: prospective.

* Studies with two groups of individuals undergoing MSA procedure.

### Endpoints

**Figure 2:**
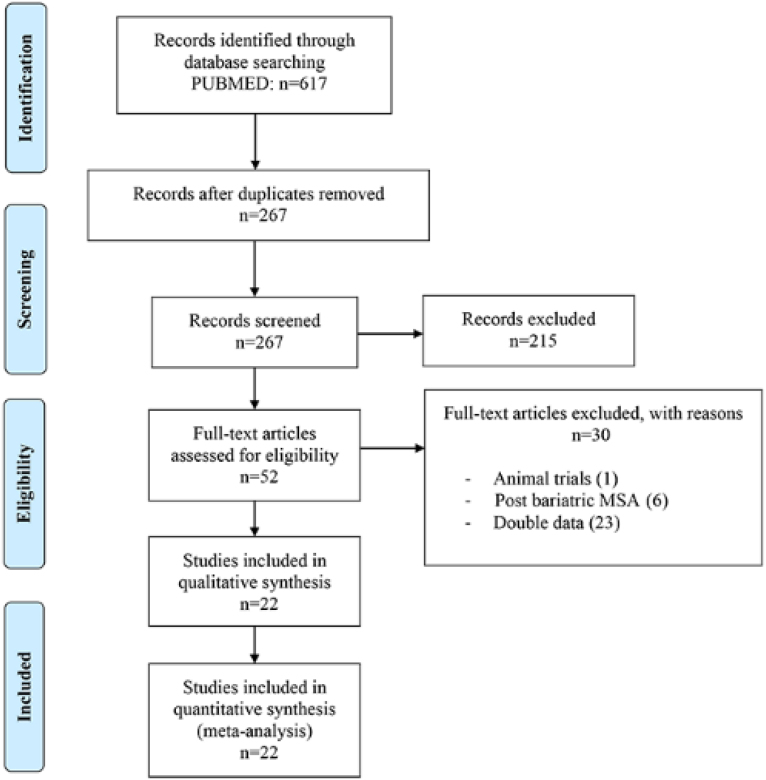
Analysis of the articles performed according to the Preferred Reporting Items for Systematic Reviews and Meta-Analysis (PRISMA).

The primary endpoint was effectiveness, which was assessed by symptom improvement, GERD-HRQL score, satisfaction rates, postoperative DeMeester score, and postprocedural use of PPIs. Secondary endpoints included: postoperative dysphagia, need for endoscopic dilation, gas-related symptoms, esophageal erosion, and device removal rates (Figure 2).

### Statistical analysis

The summary statistics were treated as independent observations and analyzed using standard methods for independent data. A meta-analysis of proportions was conducted for the following variables: symptom improvement, satisfaction, dysphagia, endoscopic dilatation, gas-related symptoms, esophageal erosion, and device removal. Statistical heterogeneity was assessed with the I2 statistic, and significance was assumed when the I2 was greater than 50%. Heterogeneity was also defined as a Cochran Q <0.10. As there was evidence of significant heterogeneity across studies, a random-effect model (DerSimonian-Laird method) was used to generate a pooled proportion with 95% confidence interval (CI) across all studies.

Average proportion of patients using PPI, DeMeester Score, and GERD-HRQL score before and after treatment were compared using a paired two-sample t-test. Logistic regression was used to model the effect of the procedure on esophageal erosion while adjusting for length of follow-up. The statistical analysis was performed using R (version 4.0.4) and R Studio (Version 1.4.1106) software. A p<0.05 was considered statistically significant in all the analyses. The study was approved by the Ethics Committee of the Institution (nº 2342346).

## RESULTS

A total of 22 studies comprising 4,663 patients with MSA device were included in the analysis. Mean age was 52.6 (39.3–64.3) years, 53% were males, and mean BMI was 27.2 (23.9–29.8) kg/m^2^. Mean operative time was 62.4 (43.2–81) minutes; 30-day overall morbidity rate was 0.7% and no mortality was reported. Mean LOS was 28.2 (5.2–53) hours. Mean follow-up across the studies was 27.3 (7–108) months. Table 1 and 2 describes the main characteristic of the studies included in the analysis.

**Figure 3:**
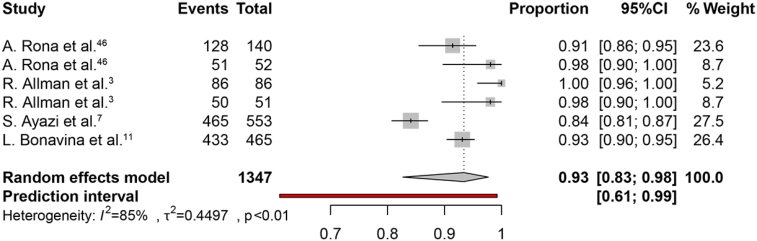
The proportion forest plot of symptom improvement.

**Figure 4:**
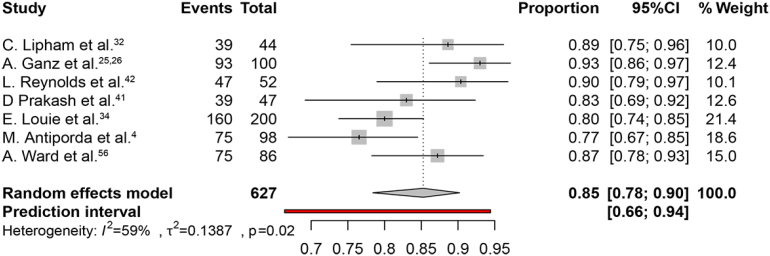
The proportion of patient satisfaction.

The weighted pooled proportion of symptoms improvement was 93% (95% CI, 83–98%) (Figure 3). The heterogeneity χ^2^ was 0.44 (p<0.01) with an I2 statistic of 85%. The weighted pooled proportion of patient satisfaction was 85% (95%CI 78–90%) (Figure 4). The heterogeneity χ^2^ was 0.14 (p<0.02) with an inconsistency (I2) statistic of 59%.

The mean DeMeester score was significantly reduced after MSA device placement (pre-MSA: 34.6 vs. post-MSA: 8.9, p=0,03). The mean GERD-HRQL score significantly improved after the procedure (pre-MSA: 25.8 vs. post-MSA: 4.4, p<0,0001). The proportion of patients taking PPIs decreased from 92.8 to 12.4% after MSA device implantation (p<0,0001).

**Figure 5:**
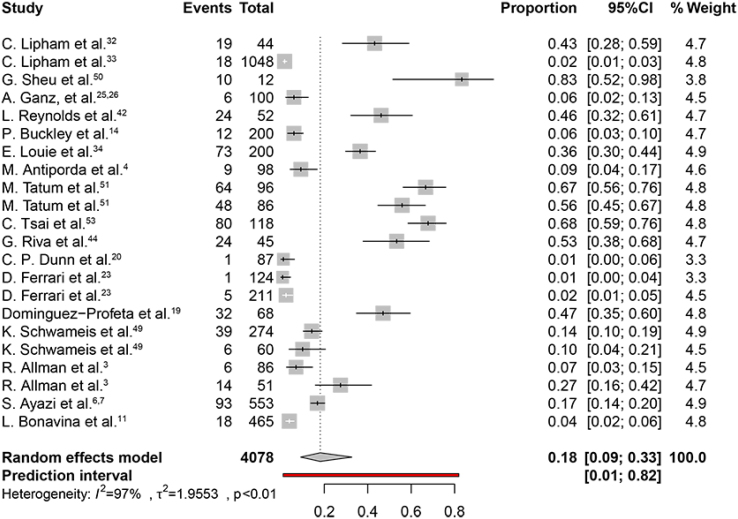
The proportion forest plot of postoperative dysphagia.

**Figure 6:**
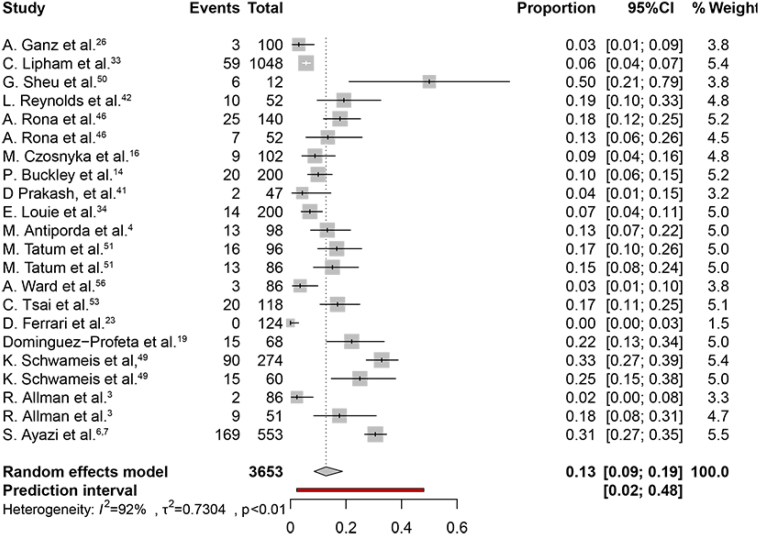
The proportion forest plot of endoscopic dilation.

The weighted pooled proportion of postoperative dysphagia was 18% (95%CI 9–33%) (Figure 5). The heterogeneity chi-squared was 1.9 (p<0.01) with an I2 statistic of 97%. The weighted pooled proportion of patients undergoing endoscopic dilatation was 13% (95%CI 9–19%) (Figure 6). The heterogeneity chi-squared was 0.73 (p<0.01) with an I2 statistic of 92%. The weighted pooled proportion of gas-related symptoms was 3% (95%CI 1–7%). The heterogeneity χ^2^ was 1.2 (p<0.01) with an I2 statistic of 76%.

The weighted pooled proportion of esophageal erosion was 1% (95%CI 0–2%). The heterogeneity chi-squared was 1.05 (p<0.04) with an I2 statistic of 50%. Follow-up time was found to be significantly associated with the odds of esophageal erosion. It was estimated that the odds of erosion increased by a factor of 1.40 per every year increase in follow-up time (odds ratio — OR 1.40, 95%CI 1.11–1.77, p=0.004). The weighted pooled proportion of patients with device removal was 4% (95%CI 3–6%). The heterogeneity χ^2^ was 0.31 (p<0.01) with an I2 statistic of 69%.

## DISCUSSION

We aimed to determine the effectiveness and safety of the MSA device placement in patients with GERD.

We found that:

- the MSA device is very effective as most patients obtain symptom relief and quality of life improvement;
- postoperative dysphagia and need for endoscopic dilation are relatively common; and
- the risk of esophageal erosion is low but increases significantly over time.

The MSA procedure is currently approved for GERD patients with indication for antireflux surgery, normal esophageal motility, BMI <35 kg/m^2^, no previous foregut surgery, and hiatal hernia <3 cm^38^. Despite these restricted indications, several studies have determined the effectiveness of the procedure. The pivotal trial, a multi-center study including 100 patients, reported a 90% reduction of GERD symptoms with 86% of patients without PPI use at two years of follow-up^3,4,7,10,11,14,16,19,20,23,25,26,34,40,41,43,45,48,50,52,55^. Similarly, a recent study including 553 patients showed that 84% of patients had at least 50% improvement in the GERD-HRQL score at a median follow-up of 10.6 months. Although our pooled analysis had a relatively short mean follow-up (27.3 months), our findings confirmed that most patients undergoing MSA implantation achieve a substantial improvement in GERD symptoms (93%) and are satisfied with the results (85%). Unfortunately, few studies showed objective data for assessment of postoperative results. DeMeester scores, however, significantly decreased in those studied with pH monitoring. In addition, few patients required PPIs after MSA implantation (12.4 %).

Dysphagia is a possible side effect of the procedure and represents one of the main indications for the MSA device removal. A previous study reported a dysphagia rate of 15.5% among 380 patients undergoing MSA placement with an overall response to the endoscopic dilatation of 68%. Only 1.8% of patients with dysphagia required device removal^6^. Another study found that the most common reason for removal was symptom recurrence (46%), followed by dysphagia (37%), and chest pain (18%)^5^. Our pooled analysis confirmed that the MSA procedure is associated with a relatively high incidence of postoperative dysphagia (18%), and a non-negligible proportion of patients (13%) required at least one endoscopic dilation.

Esophageal erosion and perforation are the main concerns after MSA device placement. In fact, "similar" type of devices such as the Angelchick prosthesis for GERD and the adjustable gastric band for obesity have been associated with these serious complications^6,54^. Salvador et al. reported two cases of severe dysphagia after MSA procedure due to migration of the device into the esophagus. The devices were safely removed endoscopically in a single step in both cases^47^. In agreement, Bona et al. concluded in 2021 that MSA devices can be safely explanted via a single-stage laparoscopic procedure associated with common antireflux procedures^9^. However, the MSA device has proven to be safe among most published studies^3-5,7,10-14,16,19,20,23,25,26,29,32-34,40,41,43,45,46,48-50,52,55^. For instance, a recent study that analyzed the manufacturer’s database reported 29 cases of erosions among 9,453 devices placed (0.3%) over four years of follow-up. Median time to erosion was 26 months, and endoscopic removal of the device was also feasible in the majority of cases^1^. The risk of erosion has been linked to the number of beads (smaller devices with small number of beads fit tightly around the esophagus). Bologheanu et al. observed that the presence of fewer than 13 beads was an independent risk factor for developing postoperative dysphagia^8^. For that reason, the MSA device with 12 beads was recently removed from the market. Our pooled analysis showed low rates of erosion (1%) and device removal (4%). Nevertheless, we found that the risk of erosion increased significantly for every year of MSA device use (OR 1.40). Therefore, considering the short follow-up of most studies, the risk of esophageal erosion should not be underestimated yet.

The Nissen fundoplication is still the mainstay of surgical treatment for GERD. Interestingly, a recent meta-analysis comparing MSA with fundoplication concluded that there were no significant differences between the procedures in terms of PPIs usage, GERD-HRQL score, dysphagia, and reoperation rates^28^. Another matched pair analysis showed that patients undergoing MSA procedure achieved similar symptom control with less gas-related symptoms, and greater ability to belch^42^. Other potential advantages of laparoscopic placement of MSA device include that it is less technically demanding, requires minimal dissection of the gastric fundus, and has no permanent anatomical alterations^31^. On the other hand, opposite to MSA procedure, the fundoplication has been used for more than 65 years and has already proven excellent long-term effectiveness^17,38,44^. High expenses and lack of coverage by many insurance companies are also drawbacks of the MSA device that should be considered.

This study has several limitations. First, several methodological design discrepancies were noted among the analyzed studies. Second, most studies included in the analysis had a short follow-up. Third, statistical heterogeneity was relevant in many of the assessed outcomes. Finally, few studies evaluated patients with postoperative pH monitoring. Future studies should include standardized diagnostic methods to allow objective and comparable assessment of outcomes.

## CONCLUSIONS

The MSA device is an effective treatment modality for GERD. Most patients undergoing MSA placement achieve symptom relief and improvement in quality of life. Postoperative dysphagia is common after the procedure. Although esophageal erosion is rare, its risk increases significantly over time. Further studies with objective assessment of results and longer follow-up are still needed.
